# Assessment of the ecological risk and mobility of arsenic and heavy metals in soils and mine tailings from the Carmina mine site (Asturias, NW Spain)

**DOI:** 10.1007/s10653-023-01848-6

**Published:** 2024-02-17

**Authors:** Rodolfo Fernández-Martínez, Noelia Corrochano, Jessica Álvarez-Quintana, Almudena Ordóñez, Rodrigo Álvarez, Isabel Rucandio

**Affiliations:** 1 Departamento de Tecnología, División de Química, Unidad de Espectroscopía, Centro de Investigaciones Energéticas, Medioambientales y Tecnológicas (CIEMAT), Av. Complutense, 40, 28040 Madrid, Spain; 2 Escuela de Ingeniería de Minas, Energía y Materiales, Dpto. de Explotación y Prospección de Minas, Universidad de Oviedo, Independencia, 13, 33004 Oviedo, Spain

**Keywords:** Arsenic, Heavy metals, Fractionation, Mobility, Abandoned mines, Ecological risk assessment

## Abstract

An evaluation of the pollution, distribution, and mobility of arsenic and heavy metals in spoil heaps and soils surrounding the abandoned Carmina lead–zinc mine (Asturias, northern Spain) was carried out. Fractionation of arsenic was performed by an arsenic-specific sequential extraction method; while, heavy metal fractionations was carried out using the protocol of the Bureau Community of Reference (BCR) (now renamed Standards, Measurements and Testing Programme). Arsenic appeared predominantly associated with amorphous iron oxyhydroxides. Among the heavy metals, lead and zinc showed high availability since significant amounts were extracted in the nonresidual fractions; whereas, chromium, copper and nickel showed very low availability, indicating their lithogenic origins. The results showed that the extractability of heavy metals in soils is influenced mainly by the presence of iron and manganese oxides as well as by pH and Eh. Multiple pollution indices, including the enrichment factor (EF), geoaccumulation index (Igeo), ecological risk index (Er) and potential ecological risk index (PERI), were used to assess the degree of soil pollution in the mine area. All results showed that lead was the key factor causing the pollution and ecological risk in the studied area, and copper, zinc and arsenic also had significant contributions. Notably, the sites at higher risk coincided with those with high availability of arsenic and heavy metals. This study provides an integrative approach that serves as a powerful tool to evaluate the metal pollution status and potential threats to the local environment of abandoned mining areas, and the results are useful for making management decisions in these areas.

**Supplementary Information:**

The online version contains supplementary material available at 10.1007/s10653-023-01848-6.

## Introduction

Long-term mining activities have led to the generation of large quantities of wastes enriched in heavy metals, which contribute to the pollution of soils and waters (Fashola et al., 2016). Exploitation of sulfide ores results in the generation of tailings rich in such mineral phases. Water runoff through mine wastes generates acidic and metal-rich leachates, which can be an environmental threat if they reach natural waters (Saria et al., 2006). The mining process involves the extraction of ores, preliminary processing of the material, disposal of both solid and liquid wastes and the transport of semiprocessed products. A variety of changes can be observed at sites close to old mining operations. Destruction of the soil structure occurs, which includes changes in the textural characteristics of the soil and acidification. The degree of soil contamination by heavy metals mainly depends on the type of ore being processed and mineral textures (Álvarez-Quintana et al., 2020). Generally, the highest impact occurs in soils located close to the source of contamination, and the impact decreases with distance (Razo et al., 2004).

The geochemical mobility of heavy metals in soils depends on the chemical forms of the metals present and their interactions with soil constituents, which, in turn, is related to the physicochemical and biological properties of the ecosystem. Therefore, understanding the speciation of elements in soils is essential to evaluate their impact and to clarify the role of the different soil phases in controlling adsorption-release processes, metal mobility and bioavailability. Interactions between ions and surface charged groups determines the mobility of heavy metals in soils. Soils are mostly composed of charged inorganic compounds, such as carbonates, silicates and iron (Fe) and manganese (Mn) oxyhydroxides. Manganese oxides can be easily mobilized by changes in environmental conditions, such as flooding and drying of the soil; whereas, well-crystallized Fe oxides must be reduced for Fe to be mobilized. In addition, several experiments have demonstrated that metal ions have a higher mobility in soils rich in organic matter. Humic and fulvic acids are the most important components of the organic fraction in determining the transport and fixation of metal ions in soils and sediments (Giacalone et al., 2005; Pagnanelli et al., 2004).

Arsenic (As) is a common constituent of many ores and appears as a major, minor or trace element in sulfides associated with waste deposits from mining activities. It is a redox-active element that is generally present in both the + 3 and + 5 oxidation states in soils. Arsenate (As(V)) is generally considered less toxic than arsenite (As(III)) (Finnegan & Chen, 2012; Molin et al., 2015). Arsenate and arsenite adsorption processes control the fate, toxicity, mobility and bioavailability of As in soils. Typically, adsorption onto clay, oxides of Fe, Mn and aluminum (Al), as well as organic matter, is the most common process for the immobilization of As compounds (Matera et al., 2003; Sadiq, 1997). Iron and Al oxides and hydroxides are the major natural minerals controlling As adsorption in soils because both As(V) and As(III) can form mono- or bidentate surface complexes with Fe oxides by inner-sphere electron transfer mechanisms (Lafferty & Loeppert, 2005; Shipley et al., 2009). Calcium carbonates play an important role in the retention and solubility of As in carbonate-rich environments. Moreover, the chemical behavior of arsenate (AsO_4_^3−^) is similar to that of phosphate (PO_4_^3−^) in soils; hence, competitive adsorption of arsenate and phosphate may occur in soils (Feng et al., 2013). Organic matter and clay contents are also two important soil factors that significantly affect the adsorption of As in soils (Álvarez-Benedí et al., 2005; Mehmood et al., 2009). Both natural and accelerated alteration of these As-bearing materials result in the release of this metalloid into waters (Zhao et al., 2021).

Sequential chemical extraction is a frequently used approach to evaluate the distribution of elements present in different chemical forms in a solid phase. In recent decades, several authors have developed specific sequential extraction methods to establish systematic elemental fractionation techniques based on metal’s mobility and environmental behavior in soils. Sequential extraction procedures (SEPs) allow for the selective fractionation of metallic elements depending on their associations with relevant phases of soil and sediments, which determine their mobility in the environment. The need for standardization led to the European Community Bureau of Reference (BCR, now renamed Standards, Measurements and Testing Programme) (Vidal & Rauret, 1993) to introduce a new three-step sequential extraction method that was later modified by Rauret et al. (1999). Since its launch, the resulting BCR sequential protocol has been applied in numerous studies and is widely accepted by the scientific community as the most suitable method to study metal fractionation in soil and sediments (Anju & Banerjee, 2010).

Most conventional SEPs are designed for the study of metals that are present as divalent cations. However, these methods are not appropriate for studying the mobility of metalloids such as As, which usually form oxyanions. To properly evaluate As fractionation, the application of specific methods that take into consideration As chemistry, is more appropriate (Larios et al., 2013).

Quantifying the ecological risk of soil pollutants is of the utmost importance for evaluating an abandoned mining site and for selecting the most suitable management procedures. Soil pollution indices, such as the enrichment factor (EF), the geoaccumulation index (Igeo) and the potential ecological risk (PERI), provide data that are ecologically and environmentally valuable to evaluate soil quality and the degree of contamination of a soil. These studies can be extrapolated to a local environment.

In the present study, soil samples from a lead (Pb)–zinc (Zn) mining site in Asturias were studied to determine the speciation of the main contaminants and their associated ecological risk several decades after the cessation of mining activities.

## Area of study

Asturias, in northwestern Spain, is a historical mining region in which energy resources such as coal, metallic ores (e.g., Au, Cu, Co, Ni, Hg, Sb, Pb and Zn, among others) and nonmetallic minerals/rocks were exploited. In terms of metal mining, only Au exploitation is now occurring in Asturias. The rest of the mines, which are now closed and abandoned, had periods of great development in the twentieth century, and they have left an environmental legacy of tailings and spoil heaps.

The Carmina mine is located in the municipality of San Martín de Oscos (*X*_UTM_ = 663,494; *Y*_UTM_ = 4,790,210, ETRS89, UTM Zone 29) in Western Asturias. Currently, this area has a low population density, with a predominance of agricultural and livestock activities. The climate is temperate (average annual temperature: 10.8 °C), with abundant rainfall throughout the year (1230 mm per year on average, of which more than half undergoes evapotranspiration).

**Fig. 1:**
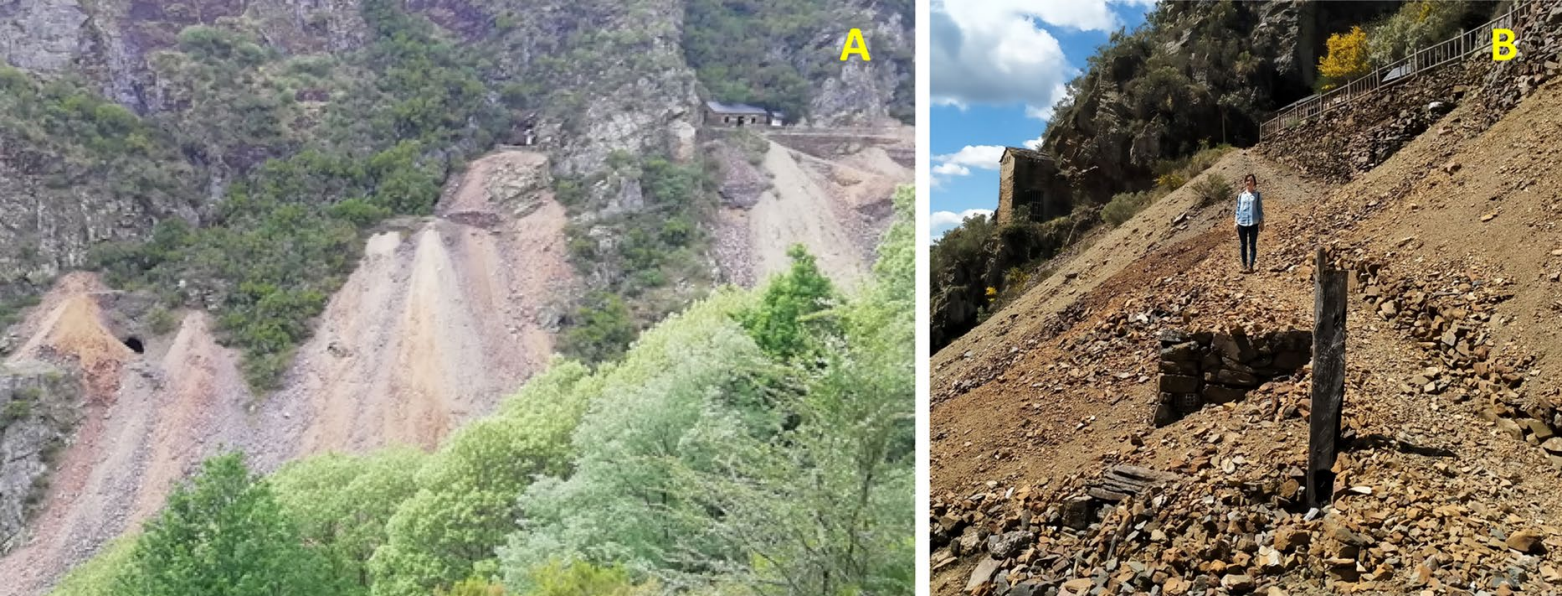
Some galleries and the hosting quartzites above the spoil heaps of the Carmina mine (**A**); Details of the spoil heaps (**B**)

**Fig. 2:**
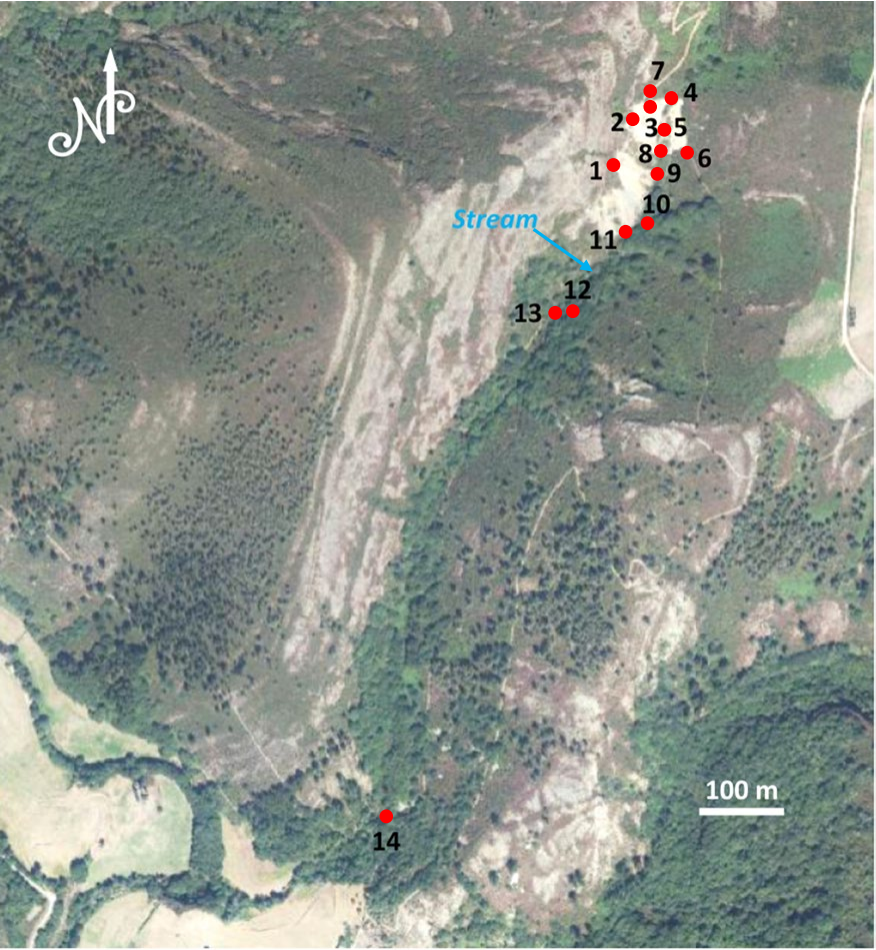
Soil sampling points location

This mine has been exploited mainly for Pb since the middle of the nineteenth century; its activity was interrupted in 1925 and was resumed in 1950. It was maintained until 1962, when mining operations ceased, mainly because of a drop in Pb prices due to toxicity (Del Valle, 2020). This mine has been recently rehabilitated and conditioned for tourist visits. The mine had 5 levels to access sphalerite-galena veins, the upper ones being at a height of 630 m a.s.l. There are four spoil heaps that contain both barren rock (mainly quartzite) that was removed to access the mineralized zones and low-grade ore (Fig. 1). It covers an area of more than 6000 m^2^ spanning both banks of a valley of a stream that is a tributary of the Agüeira River (Navia River basin) that drains the mine site (Fig. 2). There is evidence of in situ primary mineral dressing, but there are no facilities indicating metallurgical operations. The soils have reduced thickness and have low scrub, typical of areas with siliceous substrates.

The Carmina mine is geologically located in the so-called West Asturian-Leonese Zone of the Iberian Massif, close to the basal thrust of the Mondoñedo Nappe (regional-scale fracture that acts as a border between the Navia-Alto Sil and the Mondoñedo Nappe domains). Mineralization appears in the contact zone between a thick level of white and hard quartzites locally known as the Los Cabos Series and the black slates of the Luarca Formation, both of Middle Ordovician age and N–S trending, constituting the western flank of the San Martin de Oscos anticline. The paragenetic sequence of the deposit is complex: primary sulfide associations that include pyrite, pyrrhotite, cubanite, sphalerite, galena, fahlore, boulangerite and marcasite (García-Iglesias & Loredo, 1979). In terms of supergene phases, only covellite and anglesite have been found. Spatially, mineralized areas are closely related to a thin layer of garnet-rich amphibolites consisting of tremolite, spessartine, chlorite and biotite. The presence of these amphibolite layers has led some authors (García-Iglesias et al., 1985) to propose a volcano-sedimentary origin for this deposit. Mineralization is related to Hercynian fractures by filling open spaces (Garcia-Iglesias & Loredo, 1979).

## Materials and methods

### Sampling

Soil samples were collected in the areas most likely affected (those close to the mine and spoil heaps). A GPS was used to locate the sampling points. Thirteen samples were collected from the soils close to the stream, while one soil sample was collected a few meters above the stream on a part of the bank where there are no spoil heaps; this area could be considered as nonpolluted (sample 10) (Fig. 2). Alluvial soil develops preferentially on the left bank of the stream where samples 12 and 14 were taken. Each sample (1 kg) was taken at a depth of a 15 cm and kept in a labeled plastic bag. Subsequently, the samples were dried at ambient temperature for one week. Then, manual grinding in a ceramic mortar was carried out and the fraction smaller than 63 µm was recovered at the University of Oviedo facilities and sent for analysis.

### Microscopic studies

To determine the speciation of the contaminants of interest at their source, samples of mining wastes in different states of conservation were examined in the form of polished sections by means of polarization optical microscopy in reflection mode using a Leica MDLP unit. These samples, along with the soil samples, were also studied by scanning electron microscopy (SEM) using a JEOL 6610-LV unit equipped with an Inca Energy 350-Xmax50 energy dispersive X-ray spectroscopy (EDX) microanalysis module. Selected soil samples were also examined by transmission electron microscopy (TEM) using a JEOL JEM-2100 high-resolution transmission electron microscope with an EDX module. Both instruments were at the facilities of the University of Oviedo.

### Reagents, standards and apparatus

All the chemicals used were of analytical grade, and most of them were purchased from Fisher Scientific. Arsenic(III) and heavy metal standard solutions (1000 mg L^−1^, SPEX Certiprep) were employed for the preparation of working standard solutions. Ultrapure water from a Milli-Q system (18 MΩ cm, Millipore) was used throughout the experiments.

All bottles and glassware were cleaned with 0.5 M nitric acid and rinsed with ultrapure water before use to lessen potential contamination. Polypropylene centrifuge tubes (50 mL) were used for the extraction experiments.

The equipment located at the CIEMAT facilities for the sequential chemical extraction procedures consisted of a vortex mixer (Fisherbrand), an end-over-end shaker (Bunsen), a bench-top centrifuge (Eppendorf 5804), a thermostatic bath (Tectron 200, P Selecta) and a graphite block digestion system (DigiPREP Jr, SCP Science).

### Physicochemical characterization and total elemental analysis

Soil pH was measured in sample/0.01 M CaCl_2_ suspensions at a ratio of 1:5 (w/v) with a flat membrane electrode (Metrohm), according to ISO 10390:2021. The redox potential (Eh) of the samples was determined with a combined Pt ring electrode (Metrohm) in soil/water suspensions at a ratio of 1:10 (w/v). The obtained values of the oxidation reduction potential (ORP) were corrected according to the standard hydrogen electrode (SHE).

The organic matter content was determined as total organic carbon (TOC) through thermogravimetric analysis (TA Instruments SDT650), and the total carbon content (TC) was determined with an elemental analyzer (LECO CS744) by oxidative combustion with nondispersive infrared sensor detector.

The amorphous iron oxides in soils were determined after ammonium oxalate extraction, according to a method of Schwertmann (1964). The content of crystalline iron oxide was the concentration difference between total Fe and amorphous iron oxide in the soils.

Microwave-assisted acid digestion of the soil samples was carried out by an Ethos One microwave system (Milestone) according to EPA method 3051a (U.S. EPA, 2007). An amount of 0.25 g of each soil sample was digested through a mixture of 6 mL of HNO_3_, 2 mL of HCl and 4 mL of HF in a microwave at 220 °C in PTFE digestion vessels. After digestion, the PTFE capsules were poured out and heated to near dryness in the presence of 2 mL of concentrated HClO_4_ to evaporate the remaining hydrofluoric acid. The digested samples and the extracts were acidified with nitric acid, filtered through 0.45 μm cellulose paper, transferred into a volumetric flask, diluted to a final volume of 50 mL with water and stored at 4 °C for subsequent analysis.

The arsenic contents in solutions from the digested soil and SEP samples were measured by hydride generation atomic fluorescence spectroscopy (HG-AFS) using a PSA 10.055 Millennium Excalibur spectrometer. The heavy metal contents in the extracted solutions and digested samples were determined by inductively coupled plasma optical emission spectrometry (ICP‒OES) via an Agilent 5900 SVDV ICP‒OES instrument, following the EPA/200.7 method. In addition, inductively coupled plasma mass spectrometry (ICP‒MS) by a ThermoFisher iCAPRQ instrument was employed for the determination of elements with concentrations below the detection limit of ICP‒AES.

### Sequential extraction methods

Arsenic fractionation was performed using the method proposed by the authors (Larios et al., 2013) (see Table S1); while, heavy metal fractionation was evaluated through the application of the modified three-step BCR procedure (Rauret, 1998) (Table S2). The application of both methods was similar: approximately 0.5 g of each sample was accurately weighed into a 50 mL polypropylene centrifuge tube. The corresponding quantity of extractant was sequentially added; the mixture was vortexed and subjected to end-over-end rotation at 35 rpm for the time needed for each step. After each extraction, the samples were centrifuged at 5000 rpm for 30 min to separate the extract solution. A washing step was also performed with 5 mL of ultrapure water, and the washing solution was combined with the corresponding extract. The obtained solutions were filtered using 0.45 μm nylon syringe filters and diluted with ultrapure water to 50 mL. Residual fractions were obtained by digestion of the remaining solids in a graphite block digestion system with the conditions described in Table S1.

To ensure the quality of the results, both sequential extraction protocols were applied to the certified reference material CRM NIST 2710 Montana soil (with elevated trace element concentrations). All these tasks were carried out at the CIEMAT facilities.

### Data treatment by statistical analysis

The correlation matrix between the fractionated constituents and the physicochemical properties of the soils was calculated by means of Pearson’s correlation coefficients using OriginPro 7.5 software. In addition, a multivariate statistical analysis including clustering and principal component analysis (PCA) was carried out by means of IBM SPSS Statistics 27 software.

### Pollution and ecological risk assessment

Heavy metal accumulation and potential pollution were assessed by the determination of several well-established quantitative indices commonly used for the study of soils. These indices included the enrichment factor (EF), geoaccumulation index (Igeo), ecological risk index (Er) and potential ecological risk assessment (PERI).

The *Enrichment factor* (EF) for metals (Eq. 1, Table 1) allows for the detection of pollution from anthropogenic origins and the pollutant accumulation level (Akoto & Anning, 2021). The calculation of this index involves a comparison of pollutant concentration with its background value normalized by the concentration of a previously selected reference element. In the present study, titanium (Ti) was selected as the reference element (Ghrefat et al., 2011). According to Eq. (1) (Table 1), *C*_n_ is the content of the examined element in the studied soil; *B*_n_ is the reference value (local background) for the metal; Ti_sample_ is the content of the reference element (Ti) in the studied soil; and Ti_reference_ is the content of the reference element (Ti) in the reference soil sample (background).

**Table 1:** Criteria for soil classification according to the pollution indices used in this study

Index	Equation	Values	Class
EF	${\text{EF}}= \left[\frac{{\left(\frac{{C}_{n}}{{\text{Ti}}}\right)}_{{\text{sample}}}}{{\left(\frac{{B}_{n}}{{\text{Ti}}}\right)}_{{\text{reference}}}}\right]$(Eq. 1)	EF < 2	Deficiency to minimum enrichment
		2 < EF < 5	Moderate enrichment
		5 < EF < 20	Significant enrichment
		20 < EF < 40	Very high enrichment
		EF > 40	Extremely high enrichment
*I*_geo_	${I}_{{\text{geo}}}={{\text{log}}}_{2}\frac{{C}_{{\text{n}}}}{{\mathrm{1,5 }B}_{{\text{n}}}}$(Eq. 2)	Igeo < 0	Unpolluted
		0 ≤ Igeo < 1	Unpolluted to moderately polluted
		1 ≤ Igeo < 2	Moderately polluted
		2 ≤ Igeo < 3	Moderately to strongly polluted
		3 ≤ Igeo < 4	Strongly polluted
		4 ≤ Igeo < 5	Strongly to very strongly polluted
		Igeo > 5	Very strongly polluted
Er	Er = *T*_ir_ × *C*_f_ = *T*_ir_ × (*C*_s_/*B*_n_) (Eq. 3)	Er < 10	Low risk
		10 ≤ Er < 20	Moderate risk
		20 ≤ Er < 40	Considerable risk
		40 ≤ Er < 80	High risk
		Er ≥ 80	Very high risk
PERI	${\text{PERI}}= \sum_{{\text{i}}}{\text{Eri}}$(Eq. 4)	PERI < 35	Low ecological risk
		35 < PERI < 70	Moderate ecological risk
		70 < PERI < 140	Significant ecological risk
		140 < PERI < 280	Very high ecological risk
		PERI > 280	Extremely high ecological risk

The *Geoaccumulation index* (Igeo) (Mueller, 1979) was applied to evaluate the degree of metal accumulation with respect to the background level (Eq. (2), Table 1), where *C*_n_ represents the total concentration of element *n* in the soil, *B*_n_ is the background level of element *n* and 1.5 is the coefficient used to compensate for natural fluctuations and small anthropic inputs (Ho et al., 2010).

The *Ecological risk index* (Er) was calculated to comprehensively estimate the probability of harmful effects on the surrounding environment due to the presence of the studied pollutants based on heavy metal properties, environmental behavior, toxicity, and biological sensitivity (Eq. (3), Table 1) (Jiang et al., 2014). In this index, T_ir_ accounts for the toxic response factor of heavy metals. These factors for Pb, As, Zn, Cr, Ni, Cu, Fe and Mn have values of 5, 10, 1, 2, 5, 5, 1 and 1, respectively; *C*_f_ is the contamination factor; *C*_s_ represents the metal concentration in the soil; and *B*_n_ is the local background value for the corresponding metals.

*Potential ecological risk* (PERI) is expressed as the sum of the potential risks of individual metals (Eri) (Eq. (4), Table 1) (Hakanson, 1980). Classically, the PERI method includes eight pollutants, including polychlorinated biphenyls (PCBs), Hg, Cd, As, Pb, Cu, Cr and Zn. In the present study, PCBs, Hg and Cd were not considered; instead, Ni, Fe and Mn were taken into consideration. Due to the difference in pollutant types and quantity, the grading standard of heavy metal ecological risk indices was adjusted based on the types and quantity of pollutants (Yang, 2012).

## Results and discussion

### Mine waste characterization

**Fig. 3:**
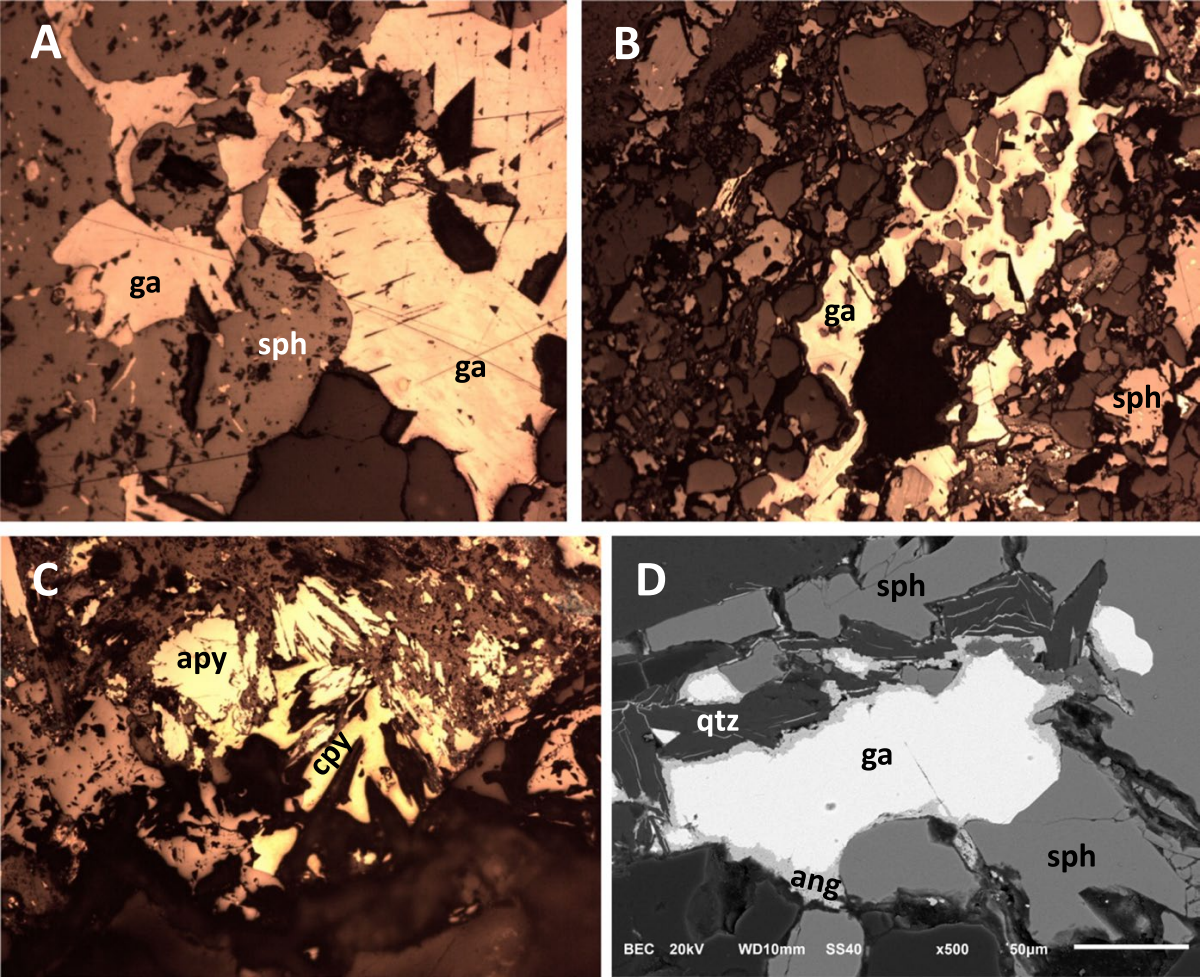
Mineral constituents of the mine wastes. **A** Galena inclusions in sphalerite denoting an early formation of Pb-sulfide. **B** Galena (ga) and sphalerite (sph) cementing silicates of the host rock. **C** Arsenopyrite (apy) and chalcopyrite (cpy), closely associated, accompanied by sphalerite. **D** Galena partially replaced by anglesite (ang) with well-preserved sphalerite and quartz (qtz). The images in Figures **A**–**C** were taken by optical microscopy in reflection mode. Figure **D** is a backscattered electron image obtained with the SEM. The horizontal framing is 0.65 mm for Figure (**A**) and 1 mm for Figures (**B** and **C**)

Spoil heaps are mainly composed of poorly mineralized rock fragments (Fig. 1). In general, primary mineral phases are in a good state of preservation due to the crystalline texture and chemically unreactive nature of the host rock (quartzites and amphibolites). The predominant sulfides are sphalerite and galena, and the latter shows clear evidence of weathering and an early formation in relation to the rest of the metallic species (Fig. 3A). EDX microanalysis of sphalerite grains demonstrates that it occurs as an Fe-rich variety known as marmatite. The epigenetic character of the mineralization results indicates that sulfides often appear as late cements between garnet grains (Fig. 3B). In some cases, a thin rim of anglesite contours galena crystals (Fig. 3C). It was not possible to verify the presence of cubanite and boulangerite in the mine wastes among the metallic minerals in this deposit (see “Area of study ”section). On the other hand, pyromorphite, chalcopyrite and arsenopyrite, which have not previously been described for the Carmina mine, were observed (Fig. 3D). Galena is the only sulfide that has crystals reaching millimeter sizes, with the rest of the sulfides showing sizes below 0.5 mm. Surface crusts of different Fe oxides (hematite, among others) are common.

### Soil physicochemical characterization

The soil parameters, including total As and heavy metal contents, are given in Table 2. Acid pH (3.3–5.6) and high Eh values (464–523 mV) were observed in all studied soil samples. Moreover, statistical analysis of the measured soil properties showed a negative correlation between pH and Eh (*r*^2^ = − 0.649 at *p* < 0.001). This might suggest that acidity occurs in the Carmina mine site as a consequence of the exposure of sulfide minerals from wastes to oxygen (Matsumoto et al., 2018). The relatively high values of TOC (3.5–25.4%) observed can be attributed to the fact that the untended areas cause a large amount of vegetal organic matter to accumulate in the steep valley, which become a part of the sampled soils. The very low calcium (Ca) contents in these samples (0.01–0.15%) reflect the noncalcareous nature of the soil mineral matter (and rocks) at this mine site.

**Table 2:** Physicochemical characterization and total elemental composition of studied soil samples

Sample	1	2	3	4	5	6	7	8	9	10	11	12	13	14
pH	3.88	5.56	4.40	3.83	4.05	3.73	5.15	3.87	3.39	4.84	4.12	3.31	3.26	4.07
Eh (mV)	523	483	513	522	502	514	464	501	494	493	495	509	514	486
TOC (%)	13.6	12.1	4.8	6.5	20.6	4.7	12	3.5	19	5.5	10.2	22.9	25.4	6.9
Al (%)	2.0	2.0	2.7	2.4	2.9	3.4	1.6	2.6	1.4	3.4	2.5	2.4	2.8	3.1
	± 0.1	± 0.8	± 0.3	± 0.4	± 0.6	± 1.0	± 0.1	± 0.1	± 0.1	± 0.2	± 0.1	± 0.1	± 0.1	± 0.1
As (mg kg^−1^)	100.8	74	115	230	55	190	111	146	49	23	52	42.6	34.7	24
	± 1.7	± 12	± 34	± 70	± 16	± 60	± 26	± 17	± 9	± 3	± 3	± 0.1	± 0.5	± 8
Ca (mg kg^−1^)	500	1500	340	240	340	430	880	390	470	380	360	480	430	70
	± 300	± 500	± 70	± 80	± 140	± 160	± 180	± 80	± 90	± 70	± 60	± 60	± 90	± 30
Cr (mg kg^−1^)	74.5	31.8	54	41	42	50	59	45	37	51	63.7	34.3	48	58
	± 0.1	± 0.4	± 19	± 16	± 5	± 30	± 5	± 2	± 2	± 4	± 0.9	± 0.5	± 2	± 5
Cu (mg kg^−1^)	358	250	640	548	560	460	98	374	110	21.4	30.1	111.6	37.6	85
	± 15	± 3	± 160	± 3	± 140	± 140	± 16	± 17	± 2	± 1.5	± 0.8	± 0.6	± 1.0	± 8
Fe (mg kg^−1^)	8.4	5.1	8.0	9.2	3.3	10.2	4.8	8–4	3.8	3.2	4.7	3.0	4.1	3.6
	± 0.3	± 0.5	± 0.1	± 0.4	± 0.1	± 0.1	± 0.9	± 0.5	± 0.2	± 0.2	± 0.1	± 0.1	± 0.5	± 0.4
Mn (mg kg^−1^)	1250	2200	3620	2000	800	3500	1150	5020	1530	913	1079	1107	565	434
	± 40	± 200	± 60	± 300	± 400	± 500	± 60	± 120	± 70	± 8	± 9	± 14	± 3	± 9
Ni (mg kg^−1^)	16.1	21.8	20.8	15	12	13	13.3	16.9	16.1	20	16.9	15.1	15.4	19.8
	± 0.5	± 1.1	± 1.0	± 6	± 4	± 3	± 0.6	± 0.8	± 1.2	± 2	± 0.3	± 1.1	± 0.6	± 1.2
Pb (mg kg^−1^)	13150	11680	39000	24500	5400	19400	3200	16500	4350	61	511.0	3163	300	1150
	± 150	± 110	± 4,000	± 160	± 90	± 300	± 400	± 300	± 140	± 17	± 1.0	± 4	± 30	± 130
Ti (mg kg^−1^)	4230	3330	4670	4600	3720	4970	4500	5400	4320	5000	4797	3610	3100	5200
	± 70	± 90	± 40	± 200	± 80	± 40	± 700	± 500	± 130	± 300	± 2	± 90	± 400	± 700
Zn (mg kg^−1^)	715	7100	1300	2300	1500	1800	935	2050	700	300	237	385	158	260
	± 13	± 130	± 100	± 700	± 300	± 600	± 100	± 20	± 30	± 20	± 5	± 3	± 8	± 20

Maximum metal concentrations were determined in the soils in the vicinity of the spoil heaps, which are definitely the main source of pollutants in the soils. However, spoil heaps are heterogeneous accumulations of residues superimposed at different times, so their geochemistry, mineralogy, and texture can be quite spatially different. This is reflected in the variability in the elemental contents of the samples; for example, the maximum in Pb is found in sample 3 and the maximum in Zn is obtained in sample 2. Very high Fe concentrations, ranging from 3 to 10%, were found in the studied soils, which is indicative of the presence of Fe-bearing minerals in the area. Luarca slates, which are more brittle than quartzites, are pyrite- and marcasite-rich and can contribute to Fe in soils. However, this Fe exists mainly in the form of oxides and silicates, not as sulfides, as evidenced in the examination of soil particles with SEM–EDX. The essential component of slates is chlorite, which is also an Fe-bearing phase. Considering the total As concentrations (23–230 mg kg^−1^), the impact of this element is as significant in this mine site as those of Pb and Zn. However, As levels remain higher than the background levels reported in the literature for soils (1–40 mg kg^−1^) (Saha et al., 2022; Salminen et al., 2005); in particular, the background level for As established by the Government of the Principality of Asturias for this region is 40 mg kg^−1^ (BOPA, 2014). Among the studied heavy metals, extremely high levels of Pb (61 mg kg^−1^ 3.9%) and Zn (158–7100 mg kg^−1^) were found in the studied soils.

### Arsenic fractionation

The total As concentrations found in the soils surrounding the Carmina mine ranged from 23 to 230 mg kg^−1^, which are markedly lower than the As levels reported in other mining areas of Asturias (e.g. Álvarez-Quintana et al., 2020; Larios et al., 2012). For example, Loredo et al. (2003) reported As concentrations ranging from 5.3 to 9116 mg kg^−1^ with a mean value of 708 mg kg^−1^ in soils from the El Terronal Hg deposit.

**Fig. 4:**
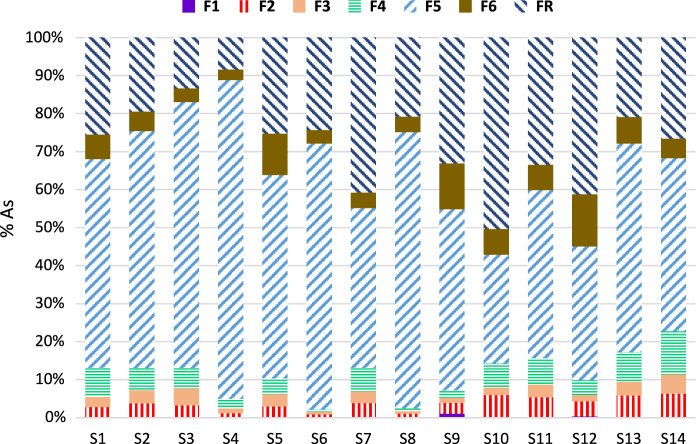
Arsenic distribution in different supporting phases in the sampled soils

The arsenic distribution patterns obtained by the specific sequential extraction method are generally similar between all the studied soil samples (Fig. 4). In the first fraction (F1), which is considered the readily available fraction and can be easily released by rainfall and other weathering processes, between 0.01 and 1.09% of the total As content was extracted. This fraction usually represents a very low proportion of As and is even negligible in soils from abandoned mines (Larios et al., 2012). The second fraction (F2) includes metal(loid)s bound to carbonates, arsenates and highly exchangeable forms (Larios et al., 2013; Patinha et al., 2004). This fraction represented 0.96–6.23% of the total As, which accounted for approximately more than 100 times the As extracted in the F1 fraction for some samples. Since the sum of fractions F1 and F2 can be considered the bioavailable As content (Du et al., 2020), the fraction of As that is strongly adsorbed onto mineral surfaces represents the bulk of the potentially mobilizable As in the area. The appreciable amounts of Ca leached in this fraction (up to 37.3% of total Ca content) can be explained by two mechanisms: (1) the presence of Ca carbonates (reported by García-Iglesias et al. (1985) in low quantities in the amphibolitic levels); (2) Ca adsorbed in clay minerals as a result of plagioclase weathering. Arsenic is likely to be present in association with these carbonates or as Ca arsenates (leachable in phosphate solutions; Matera et al., 2003). Arsenates were not directly observed, and this finding is supported by the relatively high Eh values (Eh > 450 mV; Table 2) found in the studied soils. In the third stage (F3), Al oxyhydroxides are mainly extracted by anion exchange via an NH_4_F solution (Abdala et al., 2015; Larios et al., 2013). The As recoveries obtained for the F3 fraction (0.65–5.09% of total content) indicate that As sorption onto Al oxyhydroxides is not dominant in soils from the Carmina mine area. The fourth fraction (F4) extracts As bound to organic matter. Aqueous humic acid (HA) metal complexes may in turn be strongly associated with other dissolved anions to form mixed complexes, presumably by metal-bridging mechanisms (Warwick et al., 2005). This stage is not usually considered for As-specific sequential fractionation schemes (Shiowatana et al., 2001; Wenzel et al., 2001). However, the results from this study show a significant adsorption of As onto humic matter, with recoveries ranging from 0.65 to 11.3% of the total As content, which are similar to those found in sediments from the Los Rueldos mining area (Larios et al., 2012). The presence of As bound to organic matter can be explained by the remarkable affinity of terrestrial humic matter for As species (Buschmann et al., 2006). In addition, the high Fe and Al concentrations in the studied soils can promote the formation of stable As–Fe–HA and As–Al–HA complexes (Liu et al., 2011). The F5 fraction corresponds to As bound to amorphous Fe oxyhydroxides, which are extracted by an oxalic/oxalate solution in the dark. The highest As recoveries for all the studied soils were found in the F5 fraction, ranging from 30 to 90% of the total As content, which means that As is primarily adsorbed onto amorphous Fe oxyhydroxides. In addition, a significant positive correlation was found between As extracted in the F5 fraction and amorphous Fe (similar to results from Schwertmann, 1964) (*r*^2^ = 0.770 at *p* < 0.001) (Table 3). This preferential association is expected since soils from the Carmina mine area contain large quantities of mine wastes with altered redox conditions, and sulfides are usually present in an advanced oxidation state, with Fe oxyhydroxides as coatings (Larios et al., 2012; Lim et al., 2009). The predominance of As associated with amorphous Fe oxyhydroxides has been observed in soils from other Asturian mining districts (Álvarez-Quintana et al., 2020; Larios et al., 2012). Contrary to with the observations in F5, As associated with poorly crystalline Fe oxyhydroxides was not a major fraction, accounting for only 3–13% of the total As. This is in accordance with Fe partitioning (S1). The obtained results are consistent with previous studies from other Asturian soils and sediments (Larios et al., 2012). In contrast, As linked to crystalline Fe oxyhydroxides is predominant in other ancient mining districts where mineralization consists of primary sulfide minerals, e.g., in the Talhadas area in central Portugal (Patinha et al., 2004) or in New South Wales in Australia (Bari et al., 2021). The residual fraction (FR) corresponds to As extracted from unaltered (or slightly altered) sulfide minerals and other resistant phases, such as silicates or arsenates. This fraction shows a broad range of 9.3–53.8% of total As, which means that either the As associated with unaltered waste minerals or its accumulation in secondary phases by runoff depends on the location of soils (Álvarez et al., 2006). Arsenic extracted in F6 and FR showed a significant positive correlation with crystalline Fe extracted in both fractions.

**Table 3:** Pearson’s correlation coefficient matrix for some elements and other soil parameters (*n* = 28)

	pH	Eh	TOC	Ca	Al	Mn	Fe_amorf_	Fe_cryst_
As (F1)	− 0.229	− 0.177	0.467*	0.217	− 0.731***	− 0.257	− 0.278	− 0.177
As (F2)	0.518**	− 0.179	− 0.276	0.315	− 0.394*	0.022	0.531**	0.051
As (F3)	0.432*	0.044	− 0.298	0.187	− 0.240	0.162	0.696***	− 0.027
As (F4)	0.417*	0.017	− 0.186	0.192	− 0.394*	− 0.145	0.628***	− 0.064
As (F5)	− 0.075	0.445*	− 0.527**	− 0.135	0.072	0.700***	0.770***	0.773***
As (F6)	− 0.272	0.407*	− 0.038	0.014	− 0.327	0.656***	0.533**	0.611***
As (FR)	0.163	− 0.141	− 0.367	0.192	− 0.163	0.530**	0.335	0.744***
Cu (F1)	− 0.344	0.214	0.551**	− 0.175	− 0.103	− 0.116	− 0.106	− 0.198
Cu (F2)	0.116	0.217	− 0.254	− 0.121	0.101	0.240	0.4524*	− 0.161
Cu (F3)	0.050	0.229	0.249	− 0.055	0.071	0.201	0.198	− 0.299
Cu (FR)	− 0.134	0.454*	− 0.524**	− 0.146	0.015	0.720***	0.754***	0.748***
Cr (F1)	0.377*	− 0.129	− 0.209	0.176	0.351	− 0.102	0.099	− 0.318
Cr (F2)	0.187	0.221	− 0.280	− 0.020	0.107	− 0.053	0.509**	− 0.081
Cr (F3)	− 0.076	0.317	0.213	− 0.093	− 0.175	− 0.422*	0.325	− 0.311
Cr(FR)	0.091	− 0.075	− 0.435*	− 0.390*	0.178	− 0.099	0.162	0.179
Ni (F1)	0.546**	− 0.418*	0.166	0.740***	− 0.164	− 0.362	− 0.326	− 0.366
Ni (F2)	0.102	− 0.267	0.592***	0.533**	− 0.275	− 0.612***	− 0.476**	− 0.533**
Ni (F3)	0.119	− 0.173	− 0.037	− 0.187	0.216	− 0.547**	− 0.205	− 0.639***
Ni (FR)	0.190	0.072	− 0.648***	− 0.124	0.288	0.558**	0.346	0.337
Pb (F1)	0.223	0.3745*	− 0.531**	0.101	0.072	0.575*	0.838**	0.328
Pb (F2)	0.183	0.334	− 0.394*	0.020	0.026	0.407*	0.733***	0.083
Pb (F3)	0.100	0.386*	− 0.288	− 0.003	− 0.078	0.352	0.725***	0.026
Pb (FR)	− 0.201	0.493**	− 0.509**	− 0.184	0.142	0.857***	0.699***	0.850***
Zn (F1)	0.658***	− 0.368	0.034	0.883***	− 0.270	0.032	0.043	− 0.140
Zn (F2)	0.653***	− 0.318	− 0.057	0.872***	− 0.239	0.168	0.146	− 0.017
Zn (F3)	0.619***	− 0.306	− 0.013	0.863***	− 0.269	0.023	0.088	− 0.107
Zn (FR)	− 0.145	0.359	− 0.517**	− 0.176	0.204	0.939***	0.555**	0.738***

The most significant coefficients are in bold

^*^significant at the 0.05 level (two-tailed); ^**^significant at the 0.01 level; ^***^significant at the 0.001 level

### Heavy metal fractionation

**Fig. 5:**
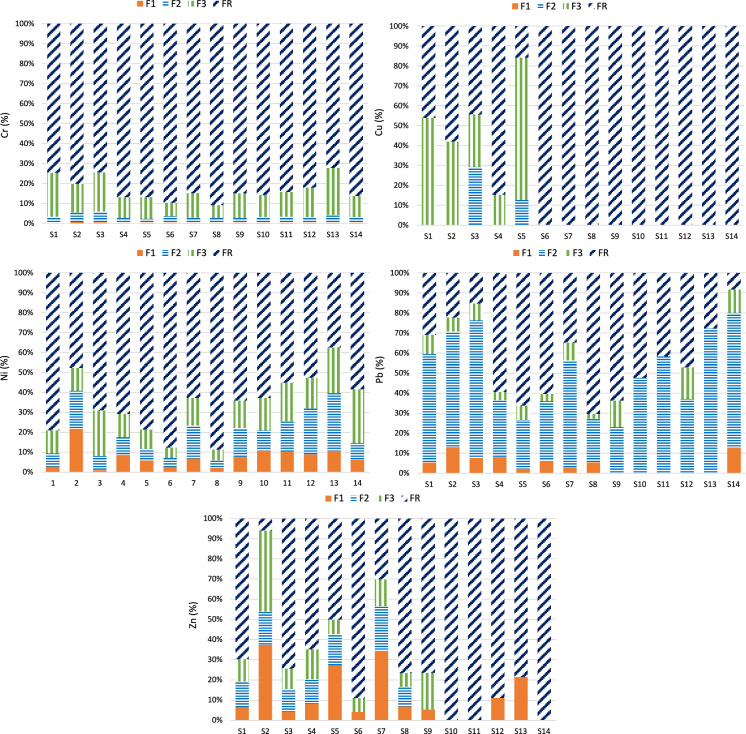
Distribution of Cr, Cu, Ni, Pb and Zn in different supporting phases of the soil samples

The results obtained for heavy metal fractionation by the BCR method are summarized in Fig. 5. In this study, the results show that the relative abundance of Cr is in the order FR > F3 > F2 > F1, and the residual fraction represents 72–91% of the total content. This indicates that Cr present in the studied soils is mainly of a lithogenic origin and is likely to be incorporated in aluminosilicate minerals and is therefore unlikely to be mobilized (Sun et al., 2019). The same occurs in other soils with similar Cr distribution patterns (Fernández et al., 2004; Nemati et al., 2011). Less than 6% of Cr was extracted in the F1 and F2 fractions, which means that Cr association with carbonates and Fe and Mn oxides is not preferred in the studied soils. However, the relatively high Cr concentrations extracted in fraction F3 revealed an important association with organic matter, which contributes to the immobilization of Cr through the reduction of Cr(VI) to Cr(III) by humic substances (Aldmour et al., 2019; Kim et al., 2019).

Copper was also predominantly present in the residual form (FR), with an average percentage of 82% of the total content, indicating that this metal is mainly associated with unaltered primary minerals. Negligible contents were observed for nonresidual fractions (F1–F3), with the exception of samples 1–5, which presented high Cu concentrations in fraction F3 (18–68% of the total content), even becoming the dominant fraction in samples 1 and 5. Copper usually exhibits a high affinity to complex with organic matter, with high stability constants (log K) showing an average value of 5.28, which is higher than those reported for other metals (Pandey et al., 2000). Hence, the high organic matter content in the studied soils may effectively contribute to the stabilization and immobilization of Cu, similar to other soils impacted by this metal (Shaheen et al., 2015).

Similar to the Cu and Cr results, the residual fraction dominates the Ni speciation in most samples. This was expected since no Ni sulfides are present: Given the very low concentrations found (< 21.8 mg kg^−1^) and in the absence of basic igneous rocks, it is expected that Ni is mainly present in low-mobile phases, such as silicates (Evans, 1989; Wali et al., 2015). However, unlike these elements, Ni is present at relevant concentrations in all the nonresidual fractions. Thus, the F1 fraction accounted for 1–22%; whereas, the F2 fraction represented 4–29% of the total Ni content. The presence of Ni contents associated with carbonates and Fe and Mn oxyhydroxides is supported by the significant positive correlations found between Ni extracted in F2 and total Ca (*r*^2^ = 0.533 at *p* < 0.01) (Table 3). Moreover, significant positive correlations were established between Ni contents in the F1 fraction and total Ca. This indicates a higher mobility of this metal, which is in accordance with observations from other soils impacted by anthropogenic activities (Iyaka, 2011). Although the association of Ni with Fe and Mn oxides is not preferred, high concentrations in this fraction are well-established in the literature (Luo et al., 2020; Žemberyová et al., 2006).

Lead was mainly present in the nonresidual fractions of the soils, indicating that this metal was potentially more available than the other studied metals. The low pH values shown in Table 2 for soil samples support the hypothesis that Pb, in addition to being more available, is also more mobile. As previously stated, the alluvial soil develops preferentially on the left bank of the stream, so the samples (12 and 14) taken on that bank have high metal content. These elevated concentrations are maintained at a certain distance from the mine (almost 1 km in the case of sample 14). The highest concentrations were found in the F2 fraction, representing 23–76% of the total Pb content, which means that Fe–Mn oxides may be the main sink for Pb in the soils from the Carmina mine area. The significant positive correlation observed for Pb contents in the F2 fraction and total Mn (*r*^2^ = 0.407 at *p* < 0.05) and amorphous Fe (*r*^2^ = 0.733 at *p* < 0.001) (Table 3) support the above statement, as well as the results of SEM–EDX analyses for mineral particles. This behavior has been observed by other authors in areas with sulfides (Rodríguez et al., 2009). In these areas, oxidation of sulfides by atmospheric exposure leads to the release of heavy metals, which tend to concentrate in secondary minerals such as Fe and Mn oxyhydroxides (He et al., 2005). The metals in Fe–Mn oxide fractions may become soluble when environmental conditions change (e.g., pH and Eh changes) (Krupadam et al., 2006). Relatively high concentrations were observed for Pb in the acid-soluble fraction for most soil samples. Since the studied soils are not calcareous, the significant Pb extracted in this fraction can be attributed to exchangeable Pb rather than associated with carbonates. The BCR protocol does not include a step to quantify the exchangeable fraction; as a result, exchangeable elements might also be included in the F1 fraction (Rauret et al., 1999). The metal content in this fraction is more available for uptake by plants, can be released with changes in the ionic strength of the medium and is sensitive to changes in pH, becoming mobile when the pH decreases (Wang et al., 2015). Although not predominant, the association of Pb with organic matter is evident since substantial concentrations were present in the F3 fraction, with recoveries ranging from 2 to 17% of the total content. The residual fraction was the second most important fraction of Pb (16–75% of total content), which was associated with unaltered sulfide minerals that could only be mobilized as a result of intense or long-term weathering (Filgueiras et al., 2002).

A wide range of Zn concentrations was found for the F1 fraction, representing 4–42% of the total Zn. In addition, a strong positive correlation with total Ca (*r*^2^ = 0.833 at *p* < 0.001) was found (Table 3). Given the high total Zn concentrations present in the studied soils, the abovementioned percentages correspond to concentrations as high as 2640 mg kg^−1^. This indicates a high availability of Zn under acidic conditions for certain samples (1–9, 12, 13), which may be more severely affected by weathering processes. In particular, this advanced weathering is marked in samples 2, 5 and 7, which are among those taken close to the spoil heaps and have the lowest S content, revealing a lower proportion of sulfides. A relatively high mobility of Zn in the soil environment (considerably more mobile than Pb) has been previously noted in the literature (Karczewska, 1996; Wilson & Pyatt, 2007). Zinc is readily adsorbed by clay minerals, carbonates, or hydrous oxides when it is in the exchangeable form (McLean and Bledsoe 1992). The zinc contents in F2 reached 1152 mg kg^−1^ in sample 2. Although the percentages of Zn associated with F2 were lower than those found for Pb for most samples, the Zn concentrations in this step were considerable. The same was found for fraction F3, which showed appreciable percentages only in the most impacted samples. The higher affinity of Zn for nonresidual fractions in the most polluted samples results in a higher potential for Zn mobilization. In contrast, FR dominates most soil samples, indicating that most of the Zn remains stable and is unable to be mobilized, as has been reported in other studies (Krupadam et al., 2006; Rodríguez et al., 2009).

### SEM/TEM examination of soil samples

**Fig. 6:**
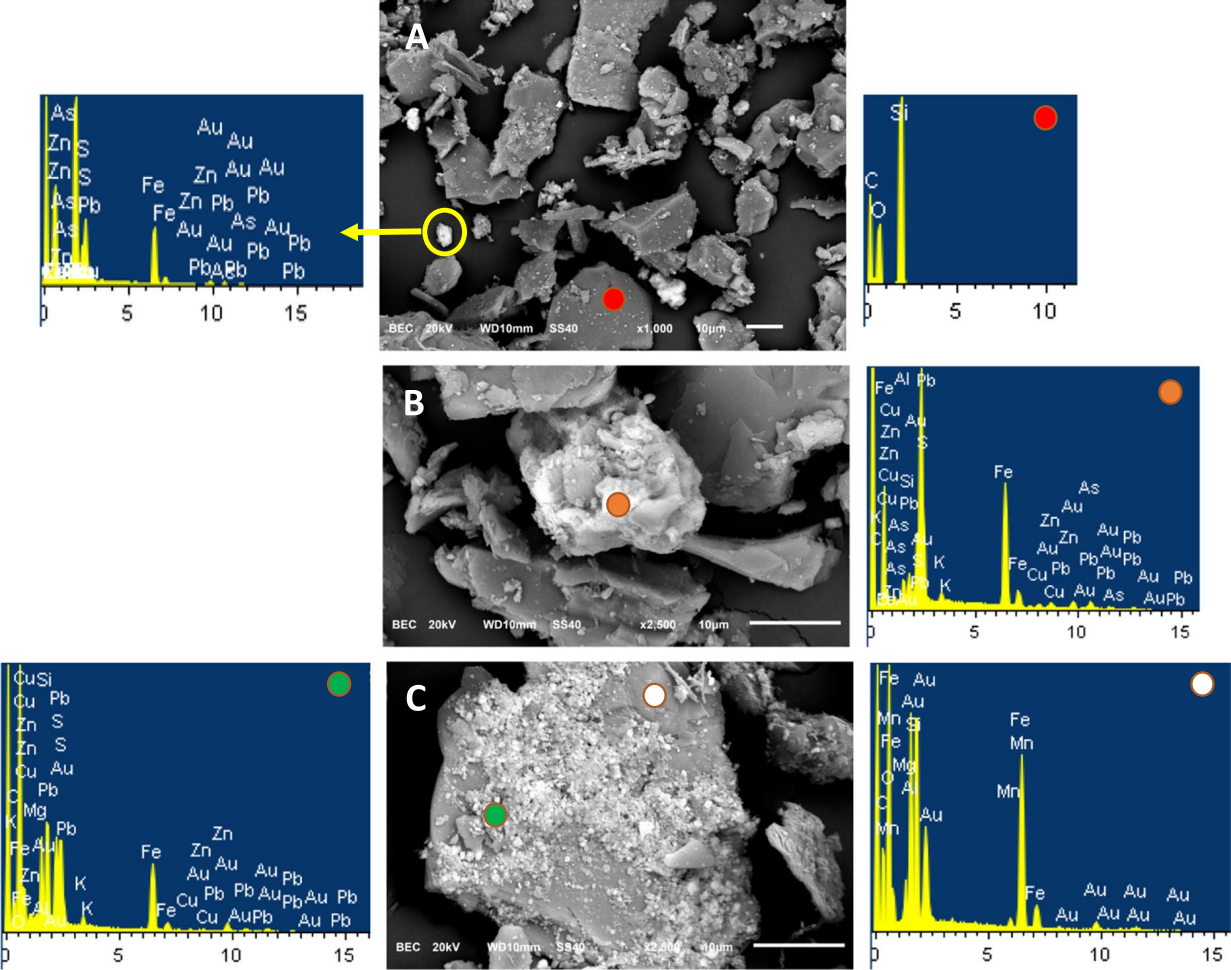
Scanning electron microscope-backscattered electrons (SEM-BCE) images. **A** Appearance of mineral particles in soil samples: angular silicates (dark tones, area analyzed and spectra indicated by a red point) and very fine particles of Fe oxides with significant amounts of Pb (6.01%) and minor quantities of As (0.15%, light-colored particles, yellow circle). **B** crystalline particle with a composition close to that of plumbojarosite (2.24% Cu, 1.29% Cu and 0.31% As, orange point). **C** crystalline Fe oxide with particulate mineral matter finely dispersed over its surface; arsenic was not detected in the supporting grain (white point), but 0.23% As and 5.85% Pb were measured over the top coating (also a Fe oxide, green point). Horizontal axis units in all spectra are keV

A representative portion of each soil sample was mounted over carbon adhesive tape and then studied by SEM. Organic matter is, in general, quantitatively important, as previously indicated. Most of the organic fraction was composed of partially degraded vegetal debris, with negligible amounts of mature organic matter. The elements As, Pb and Zn were not detectable on the organic matter surfaces with EDX microanalysis. Likewise, quartz and other aluminosilicates did not contain detectable amounts of these three elements on their surfaces (Fig. 6A). The presence of pure Al hydroxides was not observed. On the other hand, Fe oxides were common, mainly in the form of crystalline compounds, although some potentially amorphous forms (botryoidal textures and very fine-grained crusts) were also present. A compound with chemical composition close to that of plumbojarosite was quite common (Fig. 6B). EDX microanalysis indicates that the Fe oxides often contained As in the range of minor elements (0.1–1%; Fig. 6C).

**Fig. 7:**
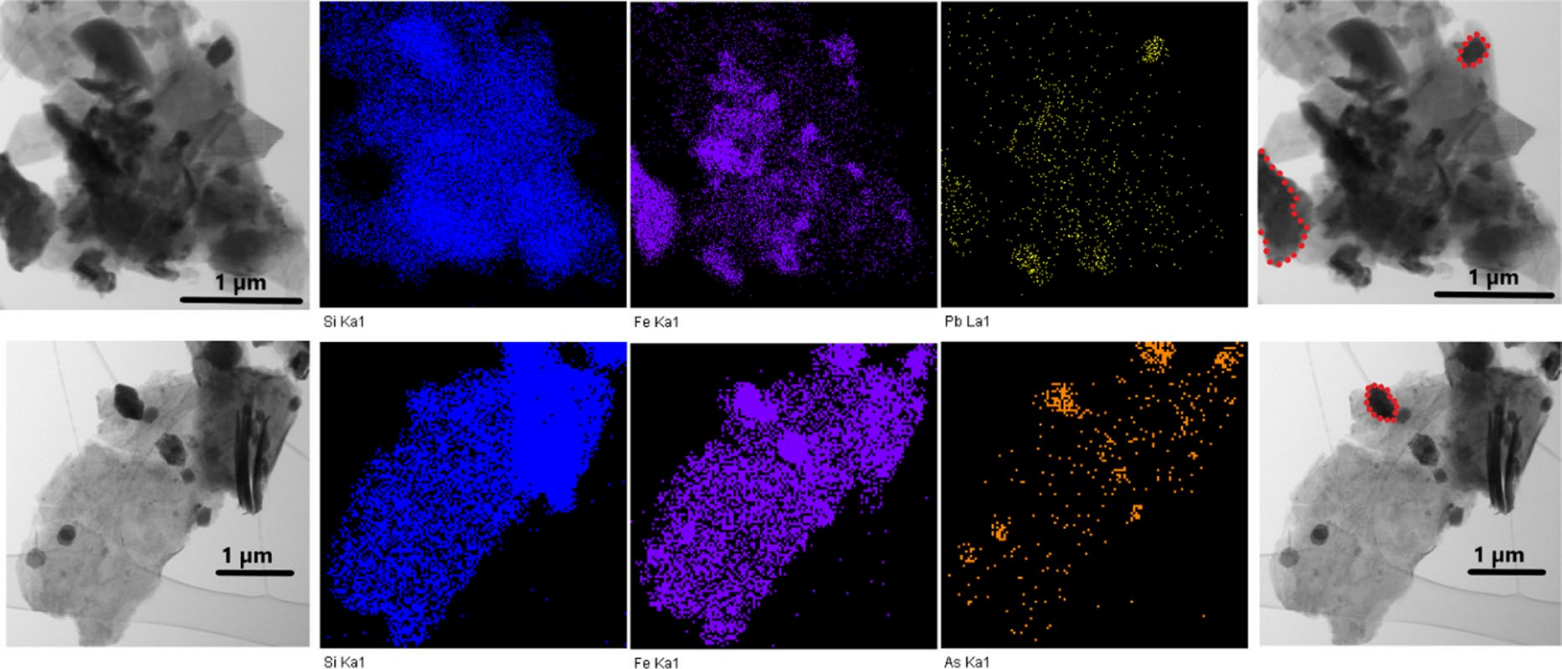
TEM images. Mapping results for Fe–Si–Pb–As in two soil particles of alkali silicates and Fe oxides. Above: Distribution of Si, Fe and Pb. Below: Distribution of Si, Fe and As. Fe oxides are indicated in red contour in the last column; the rest of the particles are silicates

Due to relatively limited magnification capacity of the SEM, elemental mapping of the mineral particles of the studied samples could not be performed. To better understand the association between Fe oxides, As and Pb (as was obtained by the employed SEPs), TEM was used. The As and Pb contents obtained on silicate surfaces were detectable as faint stippling. On the other hand, with respect to the Fe oxides, the overlap of Fe with As and Pb was usually evident, as shown in Fig. 7.

### Multivariate statistical analysis

**Fig. 8:**
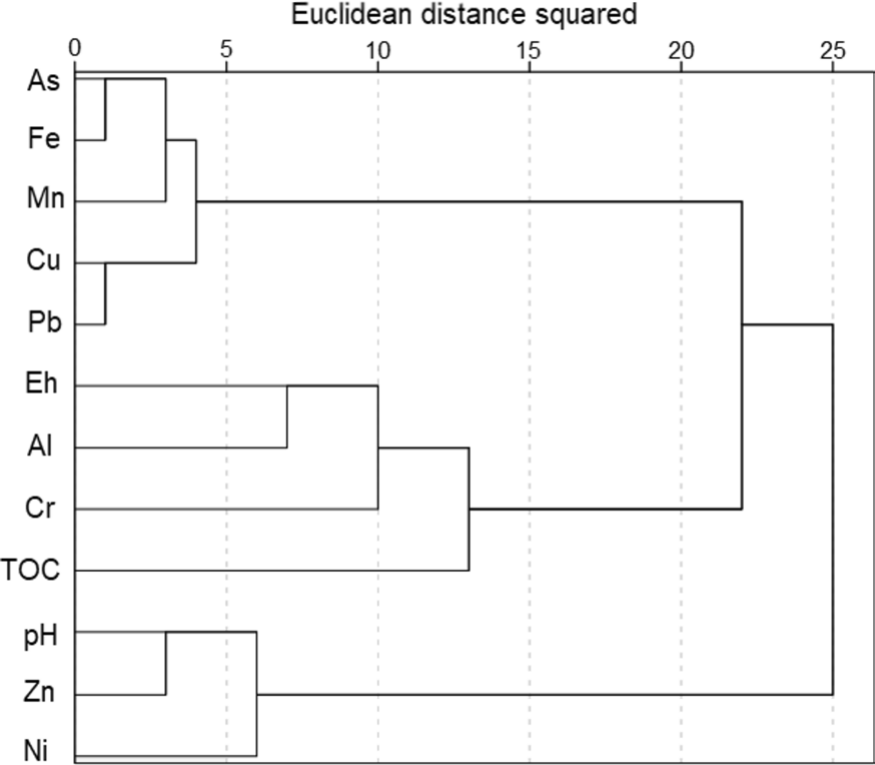
Dendrogram corresponding to statistical clustering analysis of total elemental concentrations, pH, Eh and TOC using the Ward method

The statistical study of total concentrations reveals certain associations between elements. Figure 8 shows the dendrogram obtained from the clustering analysis of select elements, pH, Eh and TOC. Three groups were found, the first consisting of As, Fe, Mn, Cu and Pb. The association between Fe and As is strong and is related to the retention of As on Fe oxides, as was confirmed in the sequential extraction and microscopy results. Thus, it is expected that the most ferriferous samples are also the most arsenical ones. Lead also shows a relationship with As, although not as close as that with Fe, since, as was seen in the fractionation study, Pb is distributed mainly between the fraction associated with Fe oxides and the residual fraction. On the other hand, there is a natural geochemical affinity between Fe and Mn; the Mn oxides consistently contain some Fe and vice versa. Copper is very close to Pb in the same group, but this metal, as well as Ni and Cr, was found in very low concentrations. The second group integrates Eh, Al, Cr and TOC. The most likely origin of Al in the soils is aluminosilicates derived from the amphibolite levels and their weathering products, and it is not linked to other metals from the first group, since metals adsorbed onto the clayey surfaces fit with the F1 of the BCR and the F2–F3 of the As SEP proposed by Larios et al. (2013), and these fractions show very low concentrations of elements in the first group. Instead, Al is linked in this group with Eh and TOC. The third group includes Zn, which is separated from the rest of the metals, since it appears predominantly in the residual fraction (in the form of the sulfide sphalerite and its weathering products), and it is not linked to Fe oxides (the lowest Zn concentrations are found in BCR F2). The distance between pH, Zn and Ni and the rest of the variables is high, but these elements are not very close to each other, as shown in the principal component analysis (PCA).

**Fig. 9:**
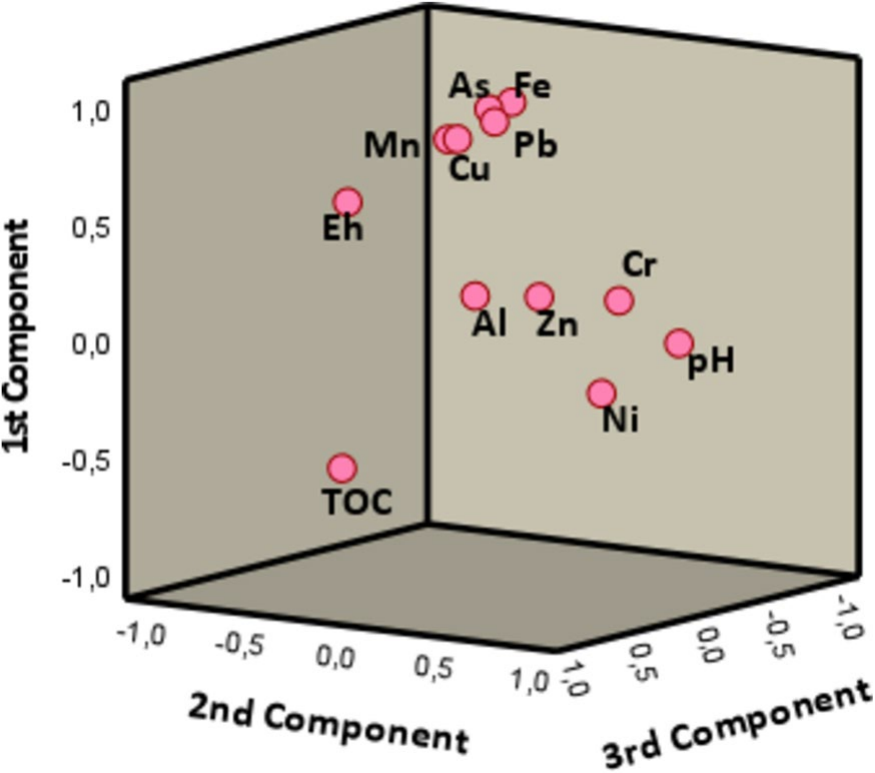
Graph of rotated components (PCA, Varimax rotation)

The results of the PCA applied to the same variables are in agreement with those found in the clustering analysis. The first three components explain 74% of the variance. Figure 9 shows the projection on the principal PCA planes. In the first component, the association of As and Pb with Fe/Mn is again demonstrated by values above 0.9. This results also corroborate that Zn does not follow this trend. Total organic carbon is not positively linked to any metal, which agrees with the low metal content of fractions F3 in BCR and F4 in the SEP proposed by Larios et al. (2013). Analogous to this, Al (related to clays) is not linked to other elements of concern due to the low metal content of fractions F1 in BCR and F2/F3 in Larios et al. (2013).

### Ecological risk assessment

Most of the samples showed no enrichment of Cr, Fe, Mn and Ni (EF < 2), even in the samples with the highest concentrations (Table 4). Although it has been established that the minimal enrichment of metals in soils can be attributed to natural factors and fluctuations in metal concentrations if EF values are lower than 2 (Weissmannová et al., 2019), it can be hypothesized that the Cr, Fe, Mn and Ni come from natural sources. The relatively higher EF values of As, Cu and Zn observed evidence the fact that the soils were enriched in these elements compared to their mean background values. The studied soils showed moderate enrichment in As and Zn and significant enrichment in Cu. The EF results indicated that the soils of the study area were highly enriched in Pb (average value of EF = 200).

**Table 4:**
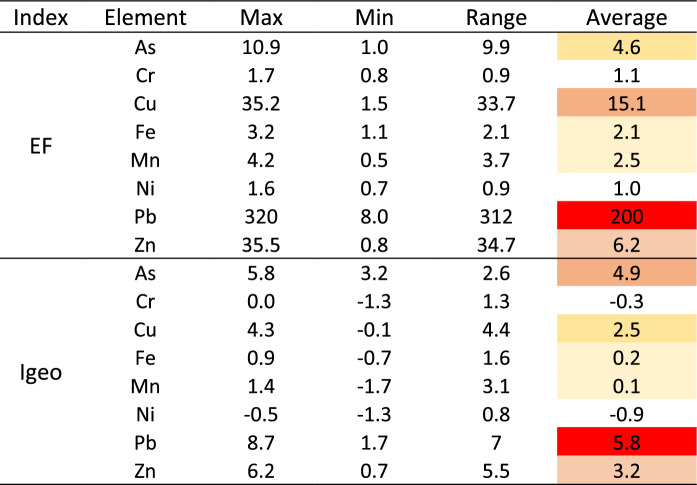
Enrichment factors (EFs) and geoaccumulation indices (Igeo) of the studied elements in soils of the area

Hereinafter, published values for EF, Igeo, Er and PERI for other Pb‒Zn mines are given for comparison with the case study. It should be noted that the extent and intensity of soil contamination due to mining waste largely depend on the nature of the waste (host rock, mineral texture, etc.), geomorphology of the area, climatic considerations and soil parameters. It is clear that not all these variables are similar for all sites. However, since the cited indices usually consider elemental concentrations relative to background levels and only include intrinsic elemental characteristics (such as toxicology) in their definition, the comparisons provide an overall picture of environmental impacts and potential consequences.

The observed mean value for EF (200) is even higher than that recently found for sulfide mine wastes at a mine site in Ghana (Akoto & Anning, 2021) and is in the same order as that found in mine tailings rich in galena, for example, in the San Quintín mining group in Spain (Martín-Crespo et al., 2015). This high value clearly indicates that the Pb enrichment in this case comes from mining waste through the process of weathering and leaching, which agrees with the high Pb availability found in the studied soils.

The results of Igeo in the Carmina mine soils exhibit various degrees of metal contamination (Table 4). The mean value of Igeo followed the order Pb > As > Zn > Cu > Fe > Mn > Cr > Ni. Chromium and Ni were in the unpolluted range; while, Fe and Mn belonged to the unpolluted to moderately polluted class. On the other hand, the studied soils are moderately to strongly polluted with Cu and strongly polluted with Zn, and As values corresponded to the strongly to very strongly polluted levels. The Igeo value for As was higher than those observed for other analogous Pb mining areas, such as San Quintín (Spain) or Zeida (Morocco) (Martín-Crespo et al., 2015; Nassiri et al., 2022). Lead from these soils showed the highest degree of contamination, with Igeo for this metal varying from 1.7 to 8.7 with a mean value of 5.8, which is similar to that observed in the San Quintín mining area (Spain) (Martín-Crespo et al., 2015). In general terms, the Igeo classification is in line with the EF classification for heavy metals as found in other studies (Fodoué et al., 2022). However, unlike EF, Igeo emphasizes As pollution in the studied soils, which is clearly in accordance with the sequential fractionation results, in which As appears to be mainly associated with secondary phases as a consequence of sulfide oxidation.

**Fig. 10:**
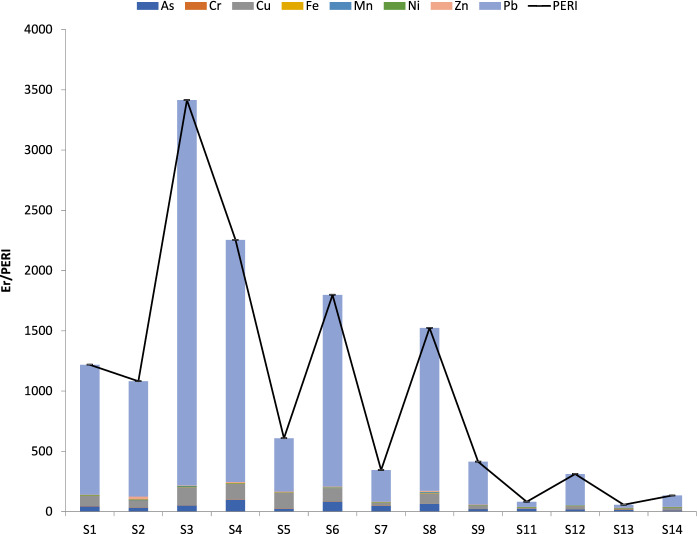
Ecological risk (Er) for each metal(loid) (bars) and integrated potential ecological risk (PERI) (black line) in the studied soils

The Er values for Cr, Fe, Mn, Ni and Zn were less than 10 for almost all samples, which implied low ecological risk (Fig. 10). This is consistent with the observed fractionation patterns of Cr and Ni, in which the residual fraction was dominant. This means that Cr and Ni are mainly present as stable primary minerals. An exception was Zn in sample 2, which poses considerable risk, and this is in accordance with the high mobility found by the application of the SEP at this site (Fig. 10). The relationship between the high Er values and the presence of heavy metals in the nonresidual fractions is well-established in the literature (Guyonnet, 2010; Kerolli-Mustafa et al., 2015). The scope of Er for As was 10.4–100.0, indicating moderate risk to very high risk. Since the As fractionation patterns were very similar for all samples, its total concentration may be the main factor explaining the risk associated with this element in the studied area. The calculated Er values showed that there is high to very high risk due to Cu contamination at most of the studied sampling points. Among all the studied pollutants, Pb was the key factor influencing the potential ecological risk, which reached an Er value of up to 3197, with a mean value of 944. These values are higher than those reported for Pb in a former Pb‒Zn mining area in northeastern Morocco (El Azhari et al., 2017).

The integrated ecological risk index (PERI) results indicate a very high risk to biota in the area due to the soil metal pollution according to the established ranges (Yang, 2012). High concentrations of these metals and As in the soil would likely lead to environmental problems such as microbial activity, biodiversity loss and poisoning through the food chain (Kars & Dengiz, 2020). Although this index combines the effect of all studied pollutants, the greatest contribution was from Pb, which is the heavy metal that most strongly exceeds its background value. The obtained values are higher than those observed in other mining districts (El Azhari et al., 2017). However, higher PERI values (reaching up to 8600) have been reported in tailings from Bibiani (North Western Ghana) (Akoto & Anning, 2021).

## Conclusions

The sequential extraction results revealed that most of the arsenic and a high proportion of heavy metals were released by oxidation and then readsorbed onto the surface of ferric hydroxides. Arsenic is readily mobilized from its original source (arsenopyrite), but it appears to be well retained in amorphous Fe (hydr)oxides. This is deduced from both SEM and SEP analyses, and was confirmed by the statistical analysis. The residual phase is quantitatively significant in some samples, which means that a portion of this metalloid is in primary and more resistant phases.

The rest of the metals studied showed different behavior. Iron was certainly of dual origin (natural and anthropic), but nickel and chromium behaved differently from copper, lead and zinc. The latter are present in the mineralization with high concentrations in the soils. The residual fraction predominates in the case of zinc and copper, which indicates that they are less easily mobilized from an environmental point of view. Lead is associated with iron oxides and is most easily released. Therefore, the role of Fe oxides from both anthropic and natural weathering origins, is environmentally relevant. Lead and zinc appear in considerable amounts bound to nonresidual fractions. Thus, they could be mobilized under appropriate environmental conditions and become bioavailable, that is, they could be easily taken up by plants growing in these soils to which they might be toxic.

This study revealed that lead poses the highest ecological threat in the study area among the studied metals. In general, the PERI results showed that both the high content of lead, and the low content or arsenic, copper and zinc posed potential risks to the environment because of their proportion in mobile fractions and their toxic response factor. One remarkable finding of this study is that, in general, the sites with high risk coincided with those with high availability of arsenic and heavy metals.

These findings provide further evidence of the potential negative effects of mine wastes on abandoned mining site environments and highlight the need for management and interventions in these areas. However, in terms of human health risk, the bioavailable fractions of As, Pb, Zn and Cu are consistently very small (except for some specific zinc samples), and this reduces risk, even when very high ecological indices for some metals are found. In addition, the mining area is very sparsely populated, so exposed sensitive receptors are scarce.

This study showed that the integration of mobility and ecotoxicity data along with multivariate statistical methods constitutes an effective tool to provide a comprehensive assessment of the impact of mine wastes on soils in surrounding areas. However, the conclusions on the impact of pollutants are limited to the soil inorganic pool. Further studies should be conducted to evaluate metal accumulation in soil biomass as plants and earthworms. Since, at present, there is no standardized method for evaluating heavy metal pollution, the methodology applied in the present work could help authorities conduct appropriate risk assessment. These findings provide further evidence of the potential negative effects of mine wastes on abandoned mining site environments. The persistence and bioavailability of heavy metals in soils from abandoned mining areas represent a possible threat to biota. Therefore, mine tailings treatment is needed to achieve waste stabilization to prevent additional weathering of sulfide minerals, which release arsenic and heavy metals. In this sense, the evaluation of green and sustainable phytoremediation strategies based on the use of native plants with phytoremediation capabilities is highly advised.

### Supplementary Information

Below is the link to the electronic supplementary material.

Supplementary file1 (DOCX 91 kb)
