# Supplementary material for: Assessment of the ecological risk and mobility of arsenic and heavy metals in soils and mine tailings from the Carmina mine site (Asturias, NW Spain)
Source: Environ Geochem Health. 2024 Feb 17;46(3):90. doi: 10.1007/s10653-023-01848-6 (PMC10874346; doi:10.1007/s10653-023-01848-6)
Supplement: Supplementary file 1 — Supplementary file1 (DOCX 91 kb) [file 10653_2023_1848_MOESM1_ESM.docx]

**Ecological risk assessment and mobility of arsenic and heavy metals in soils and mine tailings from the Carmina mine site (Asturias, NW Spain)**

Rodolfo Fernández-Martínez^a^, Noelia Corrochano^a^, Jessica Álvarez-Quintana^b^, Almudena Ordóñez^b^, Rodrigo Álvarez^b^, Isabel Rucandio^a^

*^a^Unidad de Espectroscopía, División de Química, Departamento de Tecnología, CIEMAT, Madrid, Spain*

*^b^Escuela de Ingeniería de Minas, Energía y Materiales, Dpto. de Explotación y Prospección de Minas, Universidad de Oviedo, Independencia, 13, 33004 Oviedo, Spain*

**Keywords:** Arsenic, heavy metals, fractionation, mobility, abandoned mines, ecological risk asessment

**Supplementary material**

Table S1. Arsenic sequential extraction procedure

| Step | Target phase | Extractant |
| --- | --- | --- |
| F1 | Readily soluble As | H_2_O, 30 mL, 24 h |
| F2 | Strongly adsorbed As onto mineral surfaces | 0.5 M Na_2_HPO_4_ (pH = 8), 40 mL, 8 h |
| F3 | As associated with Al oxyhydroxides | 0.5 M NH_4_F (pH = 8.2), 30 mL, 15 h |
| F4 | As bound to organic matter | 0.1 M Na_4_P_2_O_7_, 10 mL, 16 h |
| F5 | As incorporated into amorphous Fe oxyhydroxides | 0.2 M ammonium oxalate/oxalic acid (pH = 3), dark, 20 mL, 2h + 2h |
| F6 | As associated with poorly crystalline Fe (hydr)oxides | 0.2 M sodium citrate + 0.6 M sodium bicarbonate + 0.4 M ascorbic acid (pH = 8), 40 mL, 21 h |
| FR | As coprecipitated with refractory minerals | 7.5 mL HCl + 2.5 mL HNO_3_ + 2 mL HF MW assisted digestion |

Table S2. BCR procedure

| Step | Target phase | Extractant |
| --- | --- | --- |
| F1 | Exchangeable, water and acid-soluble species | 0.11 M CH_3_COOH, 20 mL, 16 h |
| F2 | Reducible species (e.g., Fe/Mn oxides, oxihydroxides) | 0.5 M NH_2_OH·HCl (+ 2.5% v/v 2 M HNO_3_), 20 mL, 16 h |
| F3 | Oxidizable species (e.g., bound to organic matter or sulfides) | 1. 8.8 M H_2_O_2_, 5 mL; Digestion in a thermostatic bath at 22˚C, 1 h; at 85˚C, 1 h 2. 8.8 M H_2_O_2_, 5 mL; Digestion in a thermostatic bath at 85˚C, 1 h 3. 1 M CH_3_COONH_4_ (pH = 2), 25 mL, 16 h |
| FR | Residual (e.g., metals bound to silicates) | 7.5 mL HCl + 2.5 mL HNO_3_ + 2 mL HF MW assisted digestion |

S3. Al, Ca, Fe and Mn fractionation by As-specific SEP
